# Beam damage in operando X-ray diffraction studies of Li-ion batteries

**DOI:** 10.1107/S160057752300142X

**Published:** 2023-03-23

**Authors:** Christian Kolle Christensen, Martin Aaskov Karlsen, Andreas Østergaard Drejer, Bettina Pilgaard Andersen, Christian Lund Jakobsen, Morten Johansen, Daniel Risskov Sørensen, Innokenty Kantor, Mads Ry Vogel Jørgensen, Dorthe Bomholdt Ravnsbæk

**Affiliations:** a Department of Physics, Chemistry and Pharmacy, University of Southern Denmark, Campusvej 55, 5230 Odense M, Denmark; b Department of Chemistry and Centre for Integrated Materials Research (iMAT), Aarhus University, Langelandsgade 140, 8000 Aarhus C, Denmark; c MAX IV Laboratory, Lund University, Fotongatan 2, SE-221 00 Lund, Sweden; d Department of Physics, The Technical University of Denmark, Fysikvej, 2880 Lyngby, Denmark; ESRF and Université Grenoble Alpes, France

**Keywords:** batteries, operando studies, beam damage, powder diffraction

## Abstract

Operando powder X-ray diffraction (PXRD) is a widely employed method for the investigation of structural evolution and phase transitions in electrodes for rechargeable batteries. Due to the advantages of high brilliance and high X-ray energies, the experiments are often carried out at synchrotron facilities. It is known that the X-ray exposure can cause beam damage in the battery cell, resulting in hindrance of the electrochemical reaction. This study investigates the extent of X-ray beam damage during operando PXRD synchrotron experiments on battery materials with varying X-ray energies, amount of X-ray exposure and battery cell chemistries. Battery cells were exposed to 15, 25 or 35 keV X-rays (with varying dose) during charge or discharge in a battery test cell specially designed for operando experiments. The observed beam damage was probed by µPXRD mapping of the electrodes recovered from the operando battery cell after charge/discharge. The investigation reveals that the beam damage depends strongly on both the X-ray energy and the amount of exposure, and that it also depends strongly on the cell chemistry, *i.e.* the chemical composition of the electrode.

## Abstract 2

An investigation is presented of X-ray beam damage during operando powder X-ray diffraction experiments on battery materials, which is a widely used technique within the field of battery science. Besides clarifying correlations between the X-ray energy and the amount of damage, it is discovered that the beam damage is surprisingly chemistry specific, *i.e.* it depends on the composition of the battery materials.

## 1. Introduction

Operando powder X-ray diffraction (PXRD) experiments on rechargeable batteries are increasingly used to study electrode materials. The technique is a powerful tool for understanding the electrode reaction mechanism in intercalation-type electrodes, such as Li-ion, and in conversion-type batteries.

X-ray diffraction was first used in an operando battery experiment in the 1970s, to follow the structural changes during Li-ion intercalation in TiS_2_ on a Phillips diffractometer operating in Bragg–Brentano reflection geometry and with Cu *K*α radiation. The PXRD was collected off the rear side (the side pointing away from the separator and counter-electrode) of the TiS_2_ electrode through a beryllium window or polyethylene bag (Chianelli *et al.*, 1978 ▸, 1979 ▸). Over the following 20 years, laboratory-based operando PXRD experiments were used for studying structural changes in intercalation materials, although the employed reflection geometry results in the electrode materials being probed primarily at the surface (Fleischmann & Mao, 1987 ▸; Samant *et al.*, 1988 ▸). Operando PXRD in transmission geometry, which probes the bulk of the operating electrode, was not feasible on in-house X-ray diffractometers due to X-ray absorption by the other cell components, *i.e.* the separator, electrolyte and counter-electrode. This experimental problem was first solved using neutrons which are highly penetrating (Latroche *et al.*, 1992 ▸). Later, Thurston and co-workers demonstrated for the first time a battery operando PXRD experiment in transmission geometry using synchrotron radiation (SR) X-rays on the X27-A diffractometer at the National Synchrotron Light Source studying an LiMn_2_O_4_–carbon cell (Thurston *et al.*, 1996 ▸). Since then, operando SR-PXRD has become state of the art for *in situ* structural determination of operating battery electrodes. An example is the finding of metastable structures in LiFePO_4_ (LFP) electrodes (Liu *et al.*, 2014 ▸), *i.e.* a non-equilibrium solid solution phase, Li_*x*_FePO_4_ (0 < *x* < 1), which forms under high current rate conditions. This phase can only be detected under dynamic conditions, as it relaxes to the end members, LFP and FePO_4_ (FP), when no current is drawn and the system is allowed to equilibrate. To enhance the use of often precious synchrotron beam time, several (often four to eight) cells are mounted and cycled simultaneously on the synchrotron diffractometer and the SR-PXRD data are collected from the cells sequentially by altering which battery cell is placed in the X-ray beam.

In operando SR-PXRD experiments a certain temporal resolution is desired, so the experiments are typically performed using relatively rapid data acquisition (of the order of 10–60 s). Thus, the high energy and high flux of SR in combination with fast area detectors are very advantageous for operando experiments. However, the SR X-rays may interfere with the cell components and hinder the electrochemical reaction or damage the materials inside the battery, as demonstrated by Borkiewicz *et al.* (2015 ▸). It is unclear whether the X-rays cause damage, or simply interfere such that the reaction is hampered locally. Hence, ‘beam-induced reaction hindrance’ may in fact be a better term for the phenomenon than ‘beam damage’. Nevertheless, the effect has traditionally been referred to as ‘beam damage’, a short term that encompasses the fact that the cell does not function as normal. We will therefore stick with this terminology.

The beam damage to the electrochemical reaction in operando SR experiments can be observed by post-cycling µPXRD mapping of the electrode, *i.e.* spatial mapping of the electrode recovered from the cell on which the operando PXRD experiment was carried out (Borkiewicz *et al.*, 2015 ▸). If beam damage has occurred, the area exposed to X-rays in the operando experiment will show a lag in the phase transformation relative to the electrochemistry and in comparison with areas not exposed to X-rays in the operando experiment. As mentioned, the beam damage is caused by *interference* between one or more of the many processes taking place in the operating cell, *e.g.* charge transfer, Li-ion diffusion, interface formation *etc.*, and/or *damage* of one or more of the components of the cell, *e.g.* the electrolyte, that is decomposed by the energy delivered by the X-ray beam and thereby loses its functionality. For interference phenomena, the extent of reaction hindrance is most likely related to the number of interfering photons, *i.e.* the dose. For damage phenomena, the reaction hindrance is more likely related to the amount of energy deposited in the damaged component, so it will be related not only to the flux and dose but also to the energy of the incoming X-rays, discussed in further detail below. The amount of energy deposited in the cell is related to the number of photons and their energy, *i.e.* it is a function of exposure time and wavelength. The exposure time is a result of the desired temporal resolution of the operando experiment, which consists of two correlated factors:

(i) Single diffractogram time resolution, *i.e.* the change in the state of charge during one frame, controlled by the exposure time versus the current, *i.e.* the C-rate, used to charge or discharge the cell. Too short an exposure results in noisy data. Too long an exposure results in averaging over too large changes in the state of charge.

(ii) Operando dataset temporal resolution, *i.e.* the number of diffractograms per unit of time, controlled by the exposure time and the number of battery cells running in parallel. Some time is ‘lost’ in motor movement and, depending on the detector type, in some overhead time, *e.g.* ‘dark exposure’. The sum of exposure time, motor time and overhead yields the overall time between measurements of each individual cell, giving the temporal resolution.

To complicate matters, the choice of wavelength has an influence on both the number of photons reaching the sample and, as mentioned, the energy deposited in the sample. At synchrotron facilities generating photons from wigglers or undulators, longer wavelengths (lower X-ray energies) typically yield higher photon fluxes (easily by several orders of magnitude), with the advantage of shorter exposure times. Additionally, the scattering angle (2θ) range becomes larger for longer wavelengths, and hence the *Q*-space or *d*-space resolution is better, *i.e.* it provides a better separation of the diffraction peaks. Lower X-ray energies might therefore be preferred, *e.g.* 15 over 35 keV. However, at lower energies relatively more energy is also absorbed by the material. For X-ray energies in the range 10–100 keV, the mass energy absorption coefficients increase linearly, roughly speaking, with decreasing energy for the elements in question (typically elements with atomic numbers 6–30) (Saloman *et al.*, 1988 ▸), *e.g.* for carbon, the mass energy absorption is a factor of ten higher at 15 keV than at 35 keV. With a higher photon flux and higher absorption factor, lowering the X-ray energy drastically increases the amount of energy dissipated in the components of the cell irradiated with the primary X-ray beam and hence increases the risk of beam damage.

Here, we present a study performed on the DanMAX beamline at the MAX IV synchrotron in Lund, Sweden, on the effect of using different X-ray energies and total exposure time (total dose) on various battery cell chemistries, as summarized in Table 1 ▸. Three cells were exposed continuously during operation with X-ray beam energies of 15, 25 and 35 keV, respectively, and a set of cells with different chemistries were exposed intermittently, *i.e.* with reduced total exposure, at 25 keV. The intermittent exposure mimics a typical operando experiment with several cells being probed in parallel. In this case, the total exposure is reduced by a factor of eight compared with continuous exposure, as four cells probed in parallel are each exposed to X-rays for 30 s every 4 min. The set of battery cell chemistries was chosen with the purpose of probing different chemistries operating at different potentials with and without Li metal. LFP in a half-cell configuration versus Li was chosen because the reaction mechanism of the Li-ion intercalation is very well documented (Ramana *et al.*, 2009 ▸; Padhi *et al.*, 1997 ▸; Liu *et al.*, 2014 ▸). The reaction mechanism is a two-phase phase transition between LFP and FePO_4_ (FP). The two end members are isostructural olivine-type structures with different lattice parameters that are nearly constant throughout the reaction. Hence, their diffractograms consist of similar patterns but with Bragg peaks separated in scattering angle, and the reaction state can therefore be unambiguously determined from the relative scales of the diffractograms throughout the whole cycle, *i.e.* all states of charge (SOC). Among the most commonly used positive electrode materials, the LFP system represents a medium/low potential (∼3.45 V versus Li/Li^+^).

LiNi_0.5_Mn_1.5_O_4_ (LNMO) in a half-cell configuration versus Li is also a well known material with two two-phase transitions during a complete charge (or discharge) (Samarasingha *et al.*, 2016 ▸). LNMO crystallizes in the spinel structure and transitions through three isostructural phases with different lattice parameters. From 0% to approximately 30% SOC (40 mAh g^−1^), phase 1 releases Li^+^ with negligible structural changes. From approximately 30% to 70% SOC (90 mAh g^−1^), phase 1 is converted to phase 2 linearly with SOC. From 70% SOC to 100%, a second phase transition occurs between phases 2 and 3, while simultaneously the amount of the remaining phase 1 is reduced. Hence, for the LNMO system, the reaction state is simplest to determine unambiguously from PXRD in approximately the 30–70% SOC regime. Among common positive electrode materials, LNMO has a high discharge potential. Furthermore, it employs different transition metals (TMs), Ni and Mn, and incorporates no P (or phosphates).

LFP in a full-cell configuration versus a graphite anode was probed to test whether potential beam damage could be associated with the Li-metal anode. A graphite half-cell versus Li was chosen as a low-voltage case without the presence of transition metals. It also served as a control experiment for the LFP–graphite full-cell. Graphite is the industry standard for many cell types and is well studied. It goes through a number of crystalline phases upon intercalation of Li that can be distinguished by PXRD (Missyul *et al.*, 2017 ▸; Boulet-Roblin *et al.*, 2017 ▸).

Through operando PXRD and subsequent electrode mapping of the cells shown in Table 1 ▸, we show that beam damage may hinder the phase evolution/electrochemical reaction in the electrode to a varying extent, depending on the beam energy and dose. Furthermore, beam damage was observed only for certain chemistries or potentials, *i.e.* the beam-damage reaction hindrance is not only beam-energy- and dose-dependent but also system specific.

## 2. Materials and method

The potential beam damage was probed by µPXRD mapping of the electrodes recovered from the battery cells used in experiments identical to typical operando PXRD experiments employing the AMPIX (Borkiewicz *et al.*, 2012 ▸) battery test cell.

For the positive electrodes composed of LFP and LMNO, electrode composites were prepared by mixing the active material (LFP KJ2, MTI Corp., USA, and LMNO TBM-129-18, Topsoe A/S, Denmark) with conductive carbon (Carbon Black Super C45, C-NERGY, Belgium) and polyvinyl­idene fluoride (PVDF, HSV900, MTI Corp., USA) as binder mater­ial in a mass ratio of 6:2:2 in acetone (97%, Sigma–Aldrich). Homogeneous slurries were obtained by shaking the mixtures for 5 min in a plastic vial containing a Teflon ball. The slurries were coated onto a glass plate and the acetone solvent evaporated. The dry coatings were scraped off the glass plate and ground into fine powders.

For graphite negative electrodes, the electrode material was scraped off a commercial electrode foil (11122, balanced, 2.4 mA h cm^−2^, Custom Cells, Germany) and used without further modification.

For all electrodes, free-standing electrode pellets were fabricated by uniaxially pressing ∼10 mg of the electrode powder composites at 15 kN for 2 min in a 7 mm-diameter pellet die to obtain electrode pellets with thicknesses of 200–300 µm. Half-cells were assembled in AMPIX battery test cells in an Ar-filled glovebox. The positive electrode pellets were placed on the glassy carbon window of the AMPIX body such that it was furthest downstream of the cell components, *i.e.* the positive electrode pellets were the last component in the cell to be exposed to the X-rays. Glass fiber filters (GF/B, Whatman) cut to 12 mm diameter were used as separators and LiPF_6_ (99.99%, Sigma–Aldrich) in 1:1 (by volume) ethylene carbonate:dimethyl carbonate (EC:DMC) as electrolyte. Metallic Li (99.9%, Sigma–Aldrich) ribbon, rolled to approximately 100 µm thickness and cut into 10 mm-diameter disks, was used as anode in the half-cells. For the full-cell, the capacities of the LFP cathode and graphite anode were not matched (balanced). Instead, the graphite anode was prepared such that the volume of the electrode pellet was within the volume restraint of the AMPIX cell, *i.e.* an approximate thickness of the anode of ∼200–300 µm is necessary for the AMPIX cell to work properly.

### 2.1. Operando setup

The AMPIX cells were mounted on the DanMAX beamline (MAX IV, Lund, Sweden) in the same manner as for a regular operando PXRD experiment. The experiments (see Table 1 ▸) were performed in two sets, with the AMPIX cells mounted on a motorized sample stage that allows for moving individual cells in and out of the primary X-ray beam. In the first set of operando experiments, LFP half-cells were exposed continuously to the unattenuated beam at 15, 25 and 35 keV, respectively, for 2.5 h each. During the 2.5 h X-ray exposure, the exposed cell was charged with a current rate of C/5 (85 mA g^−1^). In the second set of operando experiments, four cells, comprising an LFP half-cell, an LNMO half-cell, a graphite half-cell and an LFP full-cell, were mounted on the motorized sample stage in parallel. During charge at C/5, the cells were exposed alternately with 25 keV X-rays for 30 s. The stage movement time between each of the four cells was approximately 30 s, such that each cell was exposed intermittently for 30 s once during every stage cycle of 4 min.

**Figure 1:**
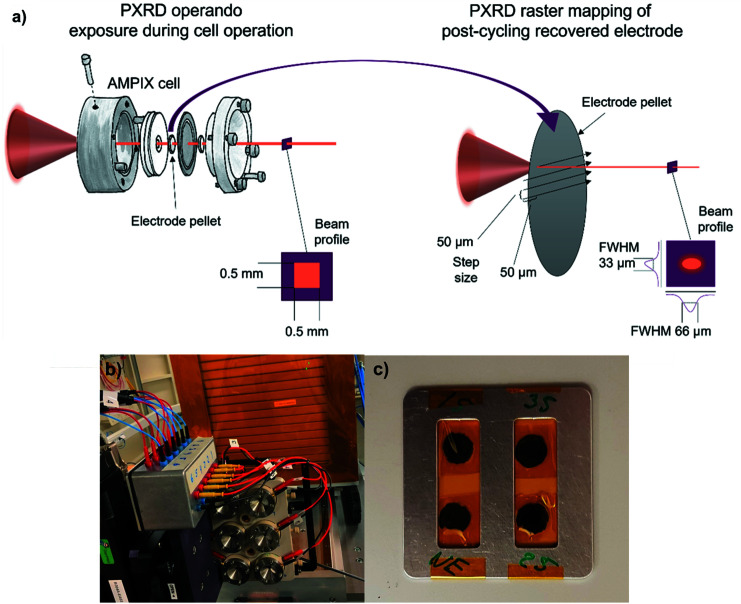
(*a*) Schematic illustrations of (left) the operando PXRD experiments exposing the AMPIX cells under operation to X-rays and (right) the µPXRD mapping experiments of the recovered electrodes. (*b*) A photograph of the operando PXRD experiment setup and (*c*) a photograph of four recovered LFP electrodes from the operando PXRD part, mounted in the sample holder in Kapton tape for the µPXRD mapping measurement.

For the operando experiments, the X-ray beam was slitted down to a 0.5 mm × 0.5 mm square profile (Fig. 1 ▸) and the scattered X-rays were collected with an area detector (PILATUS3 X 2M CdTe, Dectris). At the time of the experiment no beam monitors were available. Later the photon flux was measured with the same optics settings as used for the operando experiments. At 15 keV the flux was measured to 4 × 10^12^ photons s^−1^, at 25 keV it was measured to 1 × 10^12^ photons s^−1^ and at 35 keV it was measured to 2 × 10^11^ photons s^−1^.

For comparison, half-cells of LFP, LNMO and graphite were charged under similar conditions to those used for the operando experiments, but these cells were not exposed to X-rays during charge. Hence, no beam damage can take place in these cells.

### 2.2. µPXRD Mapping

After the operando PXRD part of the experiments, the electrodes were recovered from the AMPIX cells inside an Ar-filled glovebox and mounted between strips of Kapton tape [Fig. 1 ▸(*c*)] in an aluminium frame sample holder. In this step, the brittle electrode pellets broke apart in some cases, but their overall integrity was kept. Also, information on the rotational orientation of the pellets inside the AMPIX cell was lost in this step. The sides facing up- or downstream were maintained from the operando to the mapping experiments. However, this is of less importance since the data were acquired in transmission geometry. The recovered electrode pellets were mapped by PXRD in a raster scan with 50 µm steps using a focused beam profile of 66 µm × 33 µm (FWHM) using 35 keV X-rays (see Fig. 1 ▸). The fast scan direction of the raster scan was performed using a continuous scan. The scattered X-rays were collected with a frame rate of 25 Hz (*i.e.* 0.039 s exposure time and 0.001 s for readout) using the same area detector placed 600 mm downstream from the sample position. The scan time for each pellet was approximately 30 min for the 7 mm × 7 mm area.

### 2.3. Data analysis

The collected detector images were azimuthally integrated with the *MatFRAIA* software (Jensen *et al.*, 2022 ▸) to obtain one-dimensional intensity versus scattering angle datasets. A Rietveld model was refined against each PXRD dataset in batch mode using the *TOPAS* academic software (Coelho, 2018 ▸). For the LFP electrodes a two-phase model was set up with orthorhombic olivine LFP and FP phases. Scale factors and cell parameters *a*, *b* and *c* were refined for each phase. For the LNMO electrodes a two-phase refinement was set up with cubic spinel phases 1 and 2. Scale factors and cell parameter *a* were refined for each phase. In all refinements, a measurement of the Kapton foil from the corner where no sample was present was used as background and fitted with a refined scale parameter. The weighted residual *R*_wp_ for the fit and a measure of the agreement of the crystallographic model with the measured data, *R*_Bragg_, were computed. All frames, *i.e.* each pixel of the mapping matrices, were refined with the same starting parameters for the LFP (or LNMO) electrodes. The results from the Rietveld refinements were used to map visually the extent of reaction in each electrode pellet.

For the LFP electrodes, the FP wt% was used directly (1:1) to show the SOC of the LFP cells. For LNMO, the wt% of phase 2, *wp*_2_, was used to describe the SOC such that SOC(%) = *wp*_2_/2.8 + 50/1.4, according to Samarasingha *et al.* (2016 ▸) [see Fig. S30 of the supporting information (SI)]. This relation between the weight percent of phase 2 and the SOC (in %) is based on the following: the capacity is 140 mAh g^−1^, *i.e.* 1.4 mAh g^−1^ per percentage point in SOC. From 0 to ∼50 mAh g^−1^, the mechanism is a single-phase solid solution reaction with very little structural change. From ∼50–100 mAh g^−1^ (35–70% SOC), there is a linear change in *wp*_2_ as a function of capacity from 0 to 100 wt% (more or less). In this region the *wp*_2_ can be used to estimate the SOC. The slope of *wp*_2_ as a function of capacity is ∼2 wt% per mAh g^−1^ and the extrapolated intersection with the baseline is at ∼50 mAh g^−1^, hence *C*(mAh g^−1^) = *wp*_2_/2 + 50, where *C* is the charge in mAh g^−1^, and thus SOC(%) = *C*(mAh g^−1^)/1.4 = (*wp*_2_/2 + 50)/1.4.

The intrinsic parameters of a phase, such as the weight percent or cell parameters, diverge in areas where there is no or very little of the phase present, *e.g.* at the corners of the maps. This results in ‘noise’ when plotting these parameters as maps. These noisy areas were therefore masked. The total scale factor (sum of scale factors for the phases present in the electrode) was used to create masking maps by defining a minimum scale threshold.

## 3. Results

### 3.1. Data from the operando SRPXRD experiments

**Figure 2:**
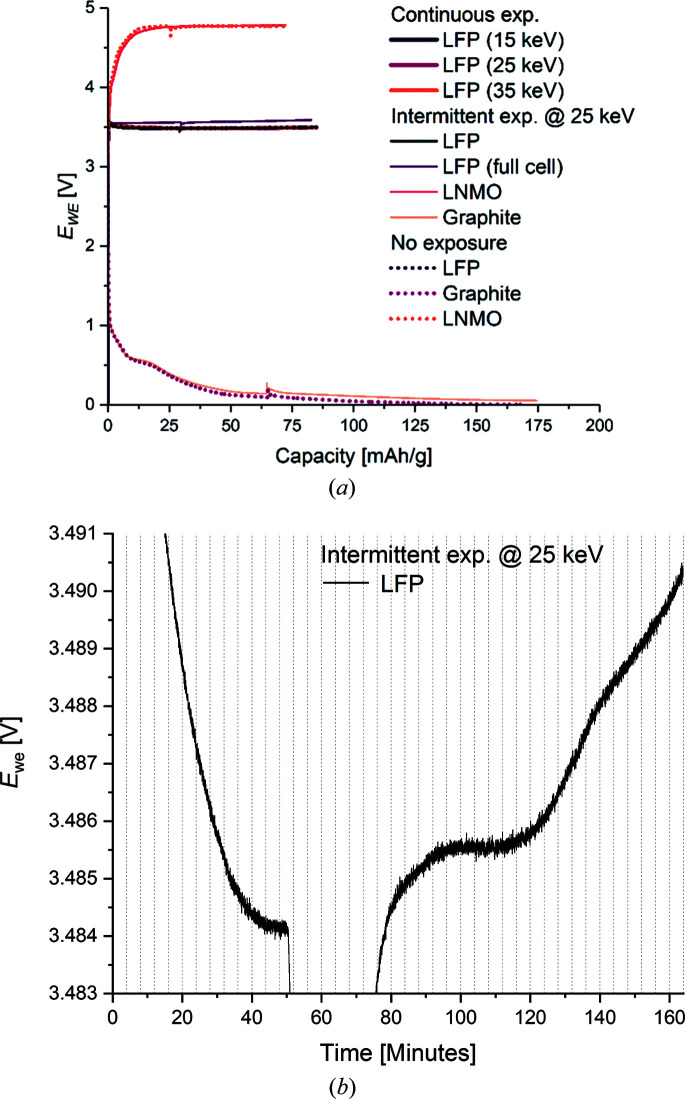
(*a*) Potential profiles for all cells under continuous exposure (thick lines), intermittent exposure (thin lines) and zero exposure to X-rays (dotted lines). (*b*) An enlargement of the potential profile for the LFP half-cell subject to intermittent X-ray exposure. The vertical dashed lines illustrate the times at which each X-ray exposure was started.

Very similar charging profiles were observed for all electrodes of each chemistry, *i.e.* for LFP, LNMO and graphite (Fig. 2 ▸). All six LFP electrodes (including the full-cell) and both LNMO electrodes were charged to 84.9 mAh g^−1^ and 71.3 mAh g^−1^, respectively, corresponding to 50% SOC for both. Both graphite electrodes of the graphite half-cells were discharged to 174.4 mAh g^−1^, corresponding to 50% SOC. As expected, a small deviation in the cell potential was observed for the full-cell compared with the half-cells. The small anomaly spikes in some of the potential profiles were due to the charge/discharge processes being paused during loss of the X-ray beam (a so-called beam dump) for ∼2 h during the operando part of the experiment. The cell potentials of those cells consequently relaxed towards their resting potentials during the pause. When the X-ray beam was re-established, the electrochemical processes were re-started and the operando experiment continued.

For each chemistry (LFP, LNMO and graphite), all the cells have similar electrochemical behavior irrespective of whether they were exposed to X-rays or unexposed, *e.g.* no changes in the cell potentials are observed for the cells during intermittent X-ray exposure. This means that there are no signs of beam damage or reaction interference observable as changes to the potential of the cell. This is not saying that beam interactions do not affect the potential in the irradiated area. The unchanged potentials may simply reflect the fact that only a small volume of the battery stack is exposed to the X-rays, *i.e.* if the potential changes in the irradiated area, this is too small to cause an observable change in the overall cell potential.

**Figure 3:**
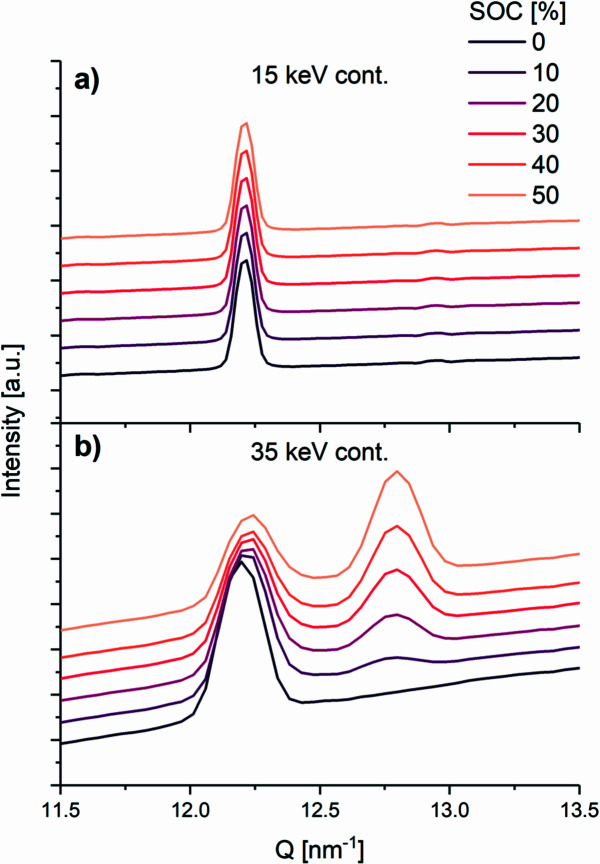
Selected regions of operando powder patterns at selected SOC showing the LFP and FP (200) reflections from (*a*) the LFP half-cell continuously exposed to 15 keV and (*b*) the LFP half-cell continuously exposed to 35 keV.

The operando PXRD data for LFP exposed continuously to the X-rays show that the diffractograms are essentially constant during the charging process when exposed to 15 keV X-rays, but when exposed to 35 keV the diffractograms change gradually with SOC (Fig. 3 ▸). Thus, for the sample exposed to 15 keV, no new diffraction peaks appear and none of the original LFP diffraction peaks disappear or lose intensity, meaning that the probed volume of LFP is not converted to FP as expected, nor is it converted to any other crystalline or amorphous phase. Hence, all the LFP in the probed volume stays intact despite the electrochemical data showing the expected process. In the cell exposed to 35 keV, as expected, LFP is gradually converted to FP in the volume probed during the operando process (see also Fig. S1 in SI), *i.e.* at 50% SOC, the LFP (200) reflection has lost half of its intensity and the (200) reflection of the FP phase has gained a similar amount of intensity. In the case of LFP and FP, comparing the intensities of two corresponding peaks, *e.g.* (200), gives quite an accurate estimate of the reaction progress because LFP and FP are isostructural with little difference in volume (<5%) and the scattering power of Li is negligible. It is also noted that the width (FWHM) of the peaks differs between the 15 and 35 keV experiments due to the difference in angular resolution.

**Figure 4:**
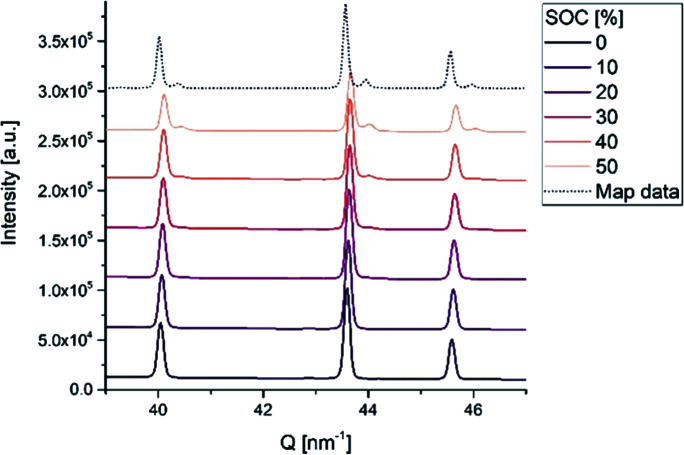
LNMO operando powder X-ray diffractograms at selected SOC (solid lines) and PXRD data from the mapping experiment (dotted line).

The operando diffraction data for the LNMO half-cell intermittently exposed to 25 keV X-rays (Fig. 4 ▸ and Fig. S2 in SI) reveal a phase transition with the onset of a new set of peaks when reaching 50% SOC. For comparison, a (random) PXRD pattern collected in the mapping experiment at a position away from the center (*i.e.* outside the area exposed to X-rays and thus not influenced by potential beam damage) is inserted at the top (dotted line). The data from the mapping experiment look very much like the operando PXRD data from the end of the electrochemical charging. Hence, the crystalline composition of the probed volume at the end of the operando experiment matches well with the general condition of the electrode pellet after recovery. This suggests that no significant beam damage affects the charge process in the LMNO electrode.

**Figure 5:**
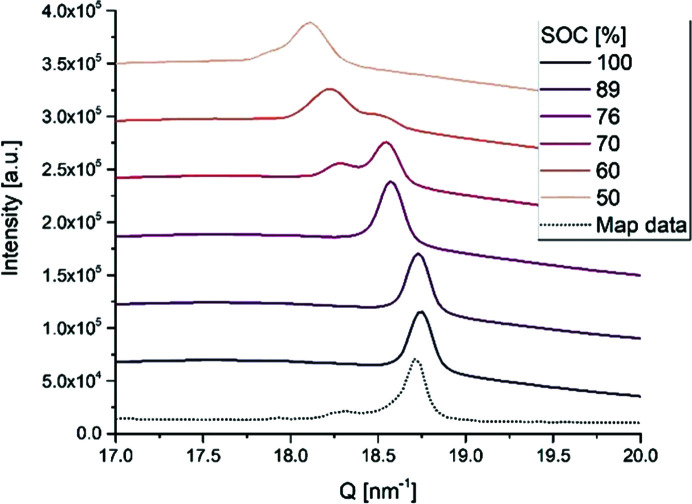
Selected regions of the operando powder patterns for graphite at selected SOC (solid lines) and PXRD data from the mapping experiment (dotted line).

The operando diffraction data from the graphite half-cell (Fig. 5 ▸ and Fig. S3 in SI) clearly show that structural changes occur during discharge with at least one phase transition during the electrochemical process. The main peak around 18–19 nm^−1^ shown in Fig. 5 ▸ is the (001) reflection that shifts towards smaller angles during discharge. This means that the interlayer distance in graphite increases with Li insertion, in agreement with earlier reports. Initially the (002) peak of the graphite phase is observed at 18.75 nm^−1^ before the electrochemical process. At the end of discharge at 50% SOC, the peak has shifted to around 18 nm^−1^ with a small shoulder on the lower side, corresponding well with the formation of an LiC_30_ phase.

We also attempted to compare the operando PXRD data with data from the mapping experiment (dotted line in Fig. 5 ▸). Unfortunately, data from the mapping experiment resemble the initial PXRD of the operando data, *i.e.* the fully charged state, which reveals that the discharged graphite is not stable in an ambient atmosphere, even though the electrode was recovered under an Ar atmosphere and sealed in Kapton tape for the mapping experiment. The electrode has probably oxidized when recovered from the AMPIX operando cell. Unfortunately, this means that it was not possible to obtain reliable information about possible beam damage in graphite from PXRD mapping. However, the operando PXRD of the graphite electrode shows that structural changes occur in the exposed volume as expected, unlike the LFP exposed continuously to 15 keV as presented above, where no reaction occurs. Hence, the graphite electrode does not seem to be affected by intermittent X-ray exposure at 25 keV.

### 3.2. Data from µPXRD mapping of the recovered electrodes

**Figure 6:**
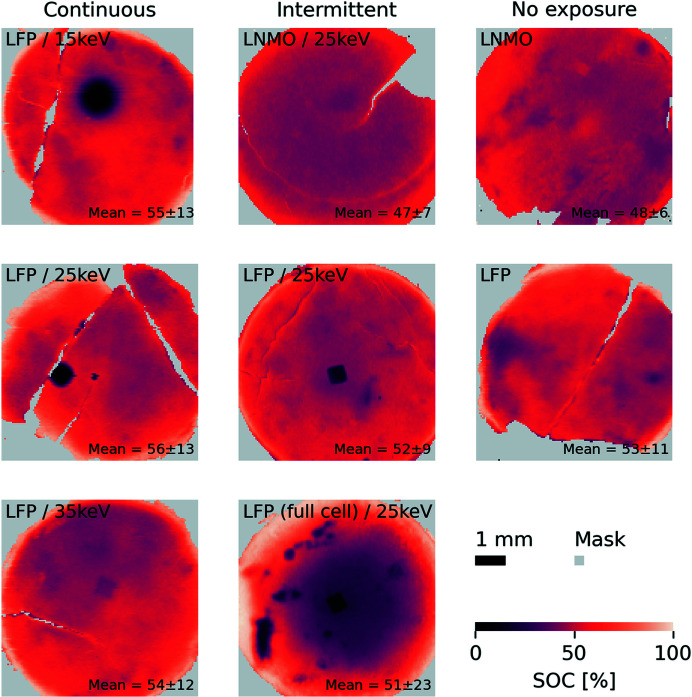
SOC maps of LFP and LNMO electrodes, based on the FePO_4_ wt% from Rietveld refinements for the LFP electrodes and on ‘phase 2’ wt% from Rietveld refinements for the LNMO electrodes. First column: LFP (half-cells) exposed continuously to (top to bottom) 15, 25 and 35 keV X-rays, respectively. Second column: electrodes from (top to bottom) LNMO and LFP half-cells and LFP full-cell, respectively, exposed intermittently to 25 keV X-rays. Third column: LNMO and LFP (half-cells) not exposed to X-rays. Mean values and standard deviations are computed from the unmasked areas.

Maps of the SOC extracted from the PXRD mapping experiments of LFP and LNMO electrodes are presented in Fig. 6 ▸ and show the state of reaction for these electrodes. Examples of the Rietveld refinements that these maps are based on are found in Fig. S4 in SI. Note that no reflections related to products forming as a result of beam interaction are observed in any of the samples. The first column of Fig. 6 ▸ (top to bottom) shows SOC maps of the LFP electrodes exposed continuously to 15, 25 and 35 keV X-rays, the second column (top to bottom) shows SOC maps of LNMO, LFP and LFP full-cell electrodes exposed to 25 keV intermittently, and the last column shows (top and middle) SOC maps from unexposed LNMO and LFP electrodes for reference. Areas of the maps with a total scale factor of the crystalline phases below 2 × 10^−8^ are masked in light gray. The mean and standard deviation of the SOC were computed from the unmasked area and are printed in the lower right-hand corner for each electrode.

All electrodes were electrochemically charged to 50% SOC, which agrees well with the average SOC determined by PXRD. The precision of the active mass fraction of the electrode is ±0.1 mg for a 10 mg electrode, *i.e.* ±1% by weight, which translates to ±1% error in the calculated cell capacities. Thus, the 1–6 percentage point overshoot in SOC for LFP and the 2–3 percentage point undershoot in SOC for LNMO as found by the PXRD mapping are generally acceptable.

In some of the SOC maps a quadratic region with an approximate size of 0.5 mm × 0.5 mm exhibiting significantly lower SOC than the average SOC is observed. The size and shape of these regions correspond well with the beam profile and thus strongly suggest that these features are caused by X-ray beam damage, and these will therefore be considered as the beam spots. Note that the beam positions may be 1–2 mm off the center of the electrode pellet because the pellet might not be perfectly aligned to the center of the AMPIX, and also because the beam position was not perfectly aligned to the center of the carbon window opening (3 mm diameter). Therefore, the beam spots can be observed rotated at a random angle and shifted a few millimetres from the center of the electrode pellets.

Maps of other refined parameters are important, but they do not provide additional information about beam damage and are therefore placed in the supporting information. Most importantly as an indication of the goodness of the fits, the weighted residuals *R*_wp_ were found to be under 10% for the full maps for both the LFP electrodes (Fig. S5 in SI) and the LNMO electrodes (Fig. S17 in SI). Also, as a descriptor of how well the crystallographic model matches the measured diffraction patterns, the Bragg *R* factors *R*_Bragg_ were generally found to be under 10% for the LNMO electrodes (Figs. S20 and S22 in SI) and under 5% for the LFP electrodes (Figs. S10 and S14 in SI). This means that the quality of the Rietveld refinements is acceptable.

The results from the series of LFP electrodes exposed continuously to the X-ray beam (Fig. 6 ▸, first column) reveal the beam-damage dependency on X-ray energy and flux. At 15 keV practically no reaction has taken place at the beam spot. From the enlargement around the beam spot in Fig. S25 in SI it is evident that the SOC has not exceeded 2% at any place in the whole region of the beam spot. This means that the LFP to FP phase transition is completely hindered by the X-ray beam. At 25 keV, the charge reaction is still lagging very much behind at the beam spot, with an average SOC of 4–5% and no areas exceeding 12% in the exposed square (Fig. S26 in SI). Hence, the reaction is delayed by about a factor of ten but not completely inhibited. In contrast to the sample exposed to 15 keV, there is no large gradient surrounding the beam spot when using 25 keV. At 35 keV a faint footprint of the beam spot with the extent of the reaction lagging slightly behind can be identified (Fig. S27 in SI). The average SOC is approximately 39% in the beam-spot square. Generally, considering the areas outside the beam spots, the extent of the reaction is not homogeneous across the pellets. This is not an effect of the beam interaction since this inhomogeneity is also observed for the unexposed pellets. Instead, this can be ascribed to the less than optimal electrode fabrication, a necessary compromise to obtain operando X-ray scattering of higher quality, compared with industrially prepared electrode coatings that are more uniform and homogeneous.

To assess the effect of reducing the total dose of X-rays by intermittent exposure, 25 keV was chosen, because at 25 keV continuous exposure the total dose did cause beam damage but did not completely inhibit the reaction, *i.e.* the beam interaction was quantitatively observable. From the experiment at 15 keV it was not possible to estimate by how much the dose should be reduced to observe a change in the reaction state within the exposed area. Note that these are ‘one shot’ experiments. Each experiment must run for the 2.5 h exposure and then be mapped with subsequent data processing before the SOC maps can be obtained and analyzed. Thus, it is not feasible to perform test shots to find the right exposure intermittency or flux within a single beam time. Hence, these experiments were performed in two separate beam times. In contrast, at 35 keV the SOC was almost not affected at the beam spot and changes as a response to the lower dose would be hard to observe. Attenuating the primary beam was considered as another way of lowering the total dose and the dose rate (flux), but, since no beam monitors were available at the time of the experiments, good control of the change in dose was not possible. Instead, intermittent exposure was employed, with the advantage that it mimics a normal operando experiment that typically entails four to eight cells running in parallel.

As observed from the SOC maps (Fig. 6 ▸, second column), the reaction in LFP intermittently exposed to 25 keV X-rays does lag behind at the beam spot, although to a lesser extent than in the continuously exposed electrode. From the enlargement of the SOC map, the average SOC in the exposed area is about 15%. Compared with the surrounding area for this electrode – which is relatively low at 40–42% SOC – 15% SOC means that the reaction is reduced by about a factor of 2–3 as a result of beam interaction. Reducing the total dose of X-rays has a clear effect in reducing the damaging impact of the beam. In this case, for an operando experiment to be feasible with LFP on the DanMAX beamline, the flux of the beam would have to be further reduced. For the LFP from the full-cell a similar picture was observed for the beam spot; the reaction lags behind by a factor of 2–3. Additionally, it is observed for this electrode employed in a full-cell that the SOC heterogeneity is significantly larger, with the standard deviation of the mean SOC being 23%, compared with 9–13% for all other LFP electrodes. There are several spots other than the beam spot with SOC lower than 10% and areas with SOC close to 100%. There is a large gradient from the center surrounding the beam spot at 20% SOC to the edge at >90% SOC. This is most likely because the AMPIX cell was not designed for the stack used in the full-cell due to two factors. First, the anode (graphite) is the same size as the cathode (LFP). Hence, misalignment of the two electrodes results in non-uniform electric fields, especially around the edges of the electrodes [by comparison, in a half-cell setup the anode (Li metal) is ‘oversized’ such that the cathode sees a more uniform electric field]. Second, the electronic conductivity in the graphite pellet and contact to the negative electrode of the AMPIX cell may be worse than for an Li-metal foil anode. This example demonstrates that the AMPIX cell is not optimal for use as a full-cell. Still, the SOC map of the LFP full-cell confirms that Li-ion cells employing LFP positive electrodes are clearly affected by the X-ray beam interaction to varying degrees depending on the X-ray energy and dose, and irrespective of the negative electrode composition. In contrast, the high-voltage LNMO electrode is not affected by the X-ray exposure to a level which is unambiguously visible. The SOC for both LNMO electrodes, exposed and unexposed, is relatively uniform with low standard deviation.

## 4. Discussion

In this small ensemble of electrode chemistries, LFP, LNMO and graphite, only the LFP electrodes were affected by beam damage, and they were affected in all cases: varying energy (even if the reaction in the LFP exposed to 35 keV was only slightly restricted), varying dose and with different counter-electrodes. This shows that beam damage (or beam-induced reaction hindrance) in battery operando X-ray experiments is system specific. This could suggest that the hampering interaction is related to the potential (∼3.5 V) of the LiFePO_4_ electrode. This might imply a process occurring at the surface or at the electrolyte–electrode interface causing the charge transfer/migration of Li ions to be restricted at medium potentials. However, the beam damage could also be related to the chemistry of the electrode, *i.e.* Fe or P, which may get ionized and hence either become inactive in the redox reaction or affect the functionality of the electrolyte. Regarding Fe, the *K* edge is at 7.1 keV, relatively close to the *K* absorption edges of Ni and Mn, at 8.3 and 6.5 keV, respectively. As the probed variation in beam energy is 20 keV, it seems unlikely that Fe should cause the large difference in the extent of beam damage between LFP and LNMO. Hence, it appears more likely that the phosphate group in LFP could be the cause of the negative impact of the X-rays.

If it is the electrolyte that is destroyed by the X-ray beam, fresh functioning electrolyte may diffuse into the irradiated volume depending on the balance between the dose rate and the diffusion rate. At low dose rates, some electrolyte would constantly be available to receive/deliver Li ions from the electrode, and hence the cells would still function at the area of incidence to some degree. On the other hand, if the dose rate is (too) high the reaction would be inhibited in the irradiated zone during the exposure time and then, with a delay, the reaction would be allowed during the unexposed period when fresh electrolyte has diffused into the affected volume. In this regard, it is interesting to note that, for the 15 keV experiment, the extent of the reaction also severely lags behind in the area surrounding the beam spot, *i.e.* there is a gradient from the edges of the beam spot extending up to 0.5 mm outwards before average SOC values are reached. The primary X-ray beam profile has a relatively sharp edge, *i.e.* at the edge the beam goes from zero to full intensity over just a few micrometres and the affected surrounding area is thus unexposed. This shows that the reaction is probably not only hampered by the direct interaction of the X-ray beam with the cell components but also suggests that whatever is damaged can diffuse into the surroundings. This supports the hypothesis of electrolyte damage.

We also note that the X-ray flux varies greatly with energy, *i.e.* one order of magnitude from 35 keV to 15 keV. This makes quantitative comparison of the beam-damage effect between the three energies difficult. A better approach would have been to attenuate the primary beam at 25 keV and 15 keV to match the flux of the 35 keV primary beam. However, flux also differs between beamlines and exact measurement of the flux at the sample position with beam monitors is rarely an option. Furthermore, users usually want to use maximum flux for fast data acquisition. Therefore, we chose not to attempt to normalize the flux with attenuators between the different energies. Instead, the total flux was reduced by intermittent exposure, mimicking a real operando experiment with four parallel cells. However, note that even though the intermittent exposure reduces the time the sample is exposed to X-rays to one eighth of that in the continuous exposure, the number of photons is only reduced by ∼40% between the 25 keV intermittent and 35 keV continuous experiments, as the flux is a factor of ∼5 higher at 25 keV than at 35 keV. Still, in the sample subjected to 35 keV continuous exposure, the reaction was hampered by only ∼5%, while 25 keV intermittent exposure restricts the reaction by more than 50%. This demonstrates that the damage is related to the energy dissipated in the cells, which differs significantly because the mass energy absorption varies with X-ray energy (Carlsson, 1985 ▸; Hubbell, 1982 ▸; Seltzer, 1993 ▸; Saloman *et al.*, 1988 ▸) (Fig. S31 in SI). Lighter elements absorb relatively less energy than heavier elements, with Fe being the dominant element responsible for energy absorption in an LFP electrode (Fig. S32 in SI). Other transition metals of the first row have similar absorption coefficients in this energy regime (below their *K* edges) (Fig. S33 in SI). Hence, the transition metals of an electrode cause the majority of energy absorption and the energy absorption will not vary significantly between electrodes of active material compounds with first-row transition metals. Energy absorption in the electrolyte and negative electrodes, Li metal or graphite, is much lower compared with the positive electrode. Still, though, these components of the cell may be less resistant to the energy delivered by the irradiating X-rays. Irrespective of photon flux, lower X-ray energies cause more beam damage than the hard X-ray regime.

## 5. Conclusions

In this work we have demonstrated that beam damage in operando SR studies of batteries is highly system specific. Beam damage was observed in operando PXRD experiments as a region of the electrode lagging behind compared with the average surroundings, and in the expected extent of reaction, *i.e.* state of charge, revealed by PXRD mapping of electrodes recovered from the cell employed in the operando experiments. The extent of the beam damage depends on the dose of the incoming X-rays and their energy (wavelength). Higher doses and lower X-ray energies result in increased beam damage. Hence, beam damage should be a concern when performing battery operando experiments utilizing SR, especially because lowering the X-ray energy at synchrotrons typically yields higher photon flux, which might be favored by the user for both spatial and temporal resolution enhancement. If no structural changes are observed through operando synchrotron X-ray scattering experiments but the electrochemistry suggests otherwise, a PXRD mapping experiment can check this relatively quickly.

Interestingly, in the ensemble of battery chemistries tested in this study (LFP, LNMO and graphite), significant beam damage was exclusively and consistently observed in cells employing an LFP electrode. Hence it seems that beam damage is correlated with the chemistry, Fe or P, or medium working potential. The results further suggest that the beam-damage mechanism can diffuse spatially in the cell, seen as a gradient of beam damage extending beyond the beam-irradiated spot into the surroundings.

Future perspectives include beam-damage studies of different chemical compositions at similar operating voltage, *e.g.* by varying the polyanion species and transition metals implemented in the electrode. This study also shows that mapping the SOC of free-standing electrode pellets from the AMPIX or similar cells is rather straightforward on the DanMAX beamline and relatively fast, with ∼1 h required per PXRD map. Beyond beam damage, the procedure and setup can also be used to probe the state of reaction heterogeneity, as seen for the full-cell, *e.g.* the influence of the thickness (total mass) of the electrode pellets, the current rate, the electrode geometry *etc*.

## Supplementary Material

Supporting Figures S1 to S33. DOI: 10.1107/S160057752300142X/fv5158sup1.pdf

## Figures and Tables

**Table 1:** Overview of experimental settings for energy, exposure and cell chemistries

	*E* (keV)	LFP half-cell	LFP full-cell	LNMO half-cell	Graphite half-cell
Continuous exposure	15	×			
	25	×			
	35	×			
Intermittent exposure	25	×	×	×	×
No exposure	–	×		×	×
