# Supplementary material for: Beam damage in operando X-ray diffraction studies of Li-ion batteries
Source: J Synchrotron Radiat. 2023 Mar 23;30(Pt 3):561–70. doi: 10.1107/S160057752300142X (PMC10161878; doi:10.1107/S160057752300142X)
Supplement: Supplementary file 1 [file s-30-00561-sup1.pdf]

JOURNAL OF SYNCHROTRON RADIATION

Volume 30 (2023)

Supporting information for article:

Beam damage in operando X-ray diffraction studies of Li-ion batteries

Christian Kolle Christensen, Martin Aaskov Karlsen, Andreas Østergaard Drejer, Bettina Pilgaard Andersen, Christian Lund Jakobsen, Morten Johansen, Daniel Risskov Sørensen, Innokenty Kantor, Mads Ry Vogel Jørgensen and Dorthe Bomholdt Ravnsbaek

Operando PXRD data

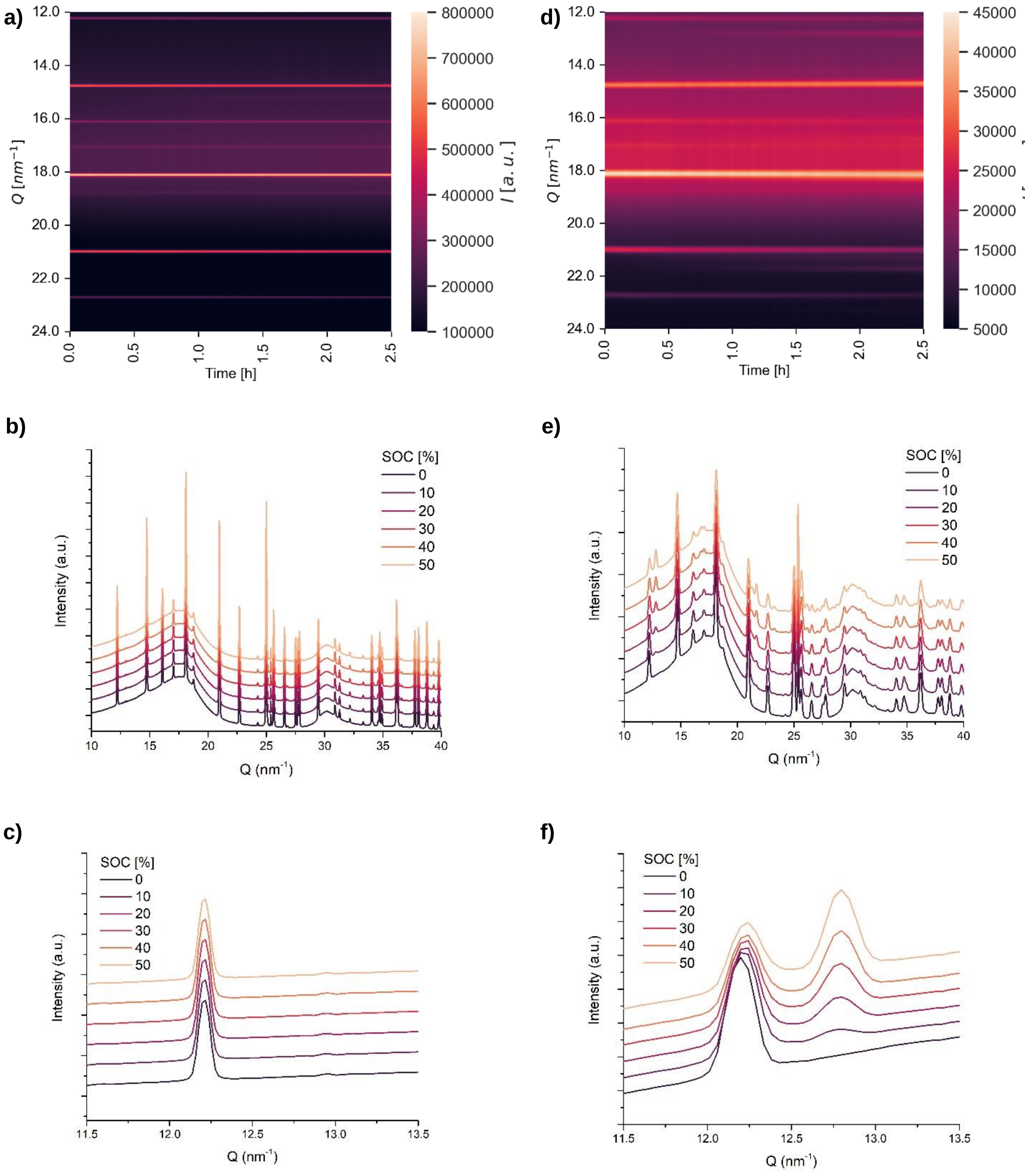

Figure S1 The “overview” PXRD from a) the LFP cell continuously exposed to 15 keV, and d) the LFP cell continuously exposed to 35 keV, PXRDs at selected SOC from b) LFP cell continuously exposed to 15 keV, and e) LFP cell continuously exposed to 35 keV, and PXRDs at selected SOC zoomed around the LFP and FP (200) reflection from c) LFP cell continuously exposed to 15 keV, and f) LFP cell continuously exposed to 35 keV.

a)

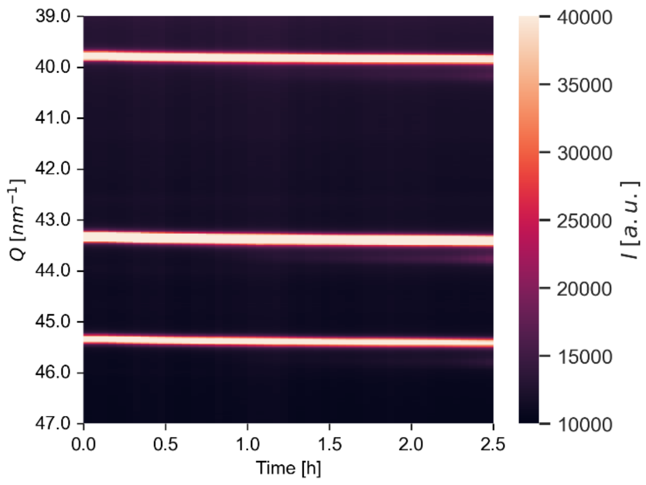

b)

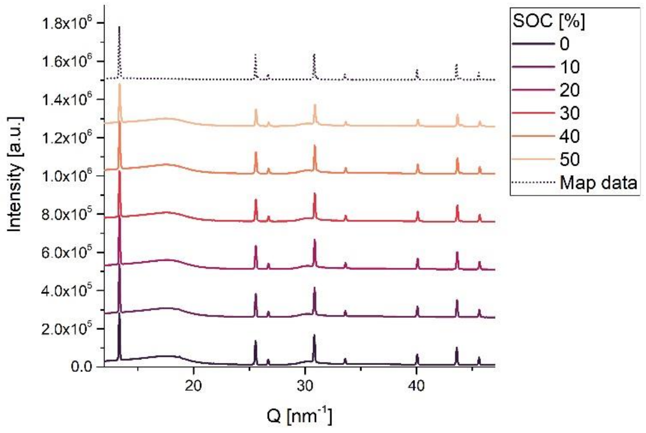

c)

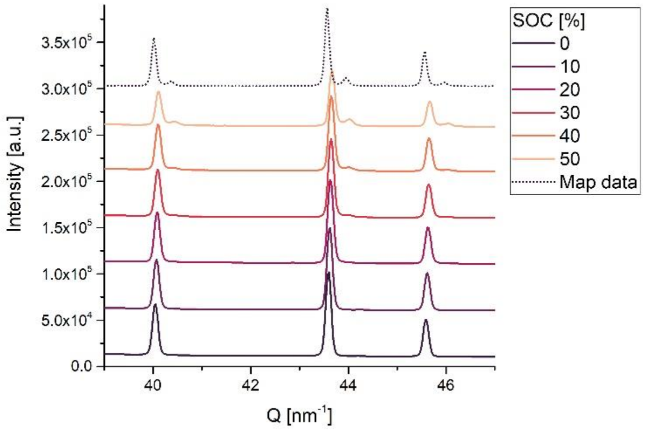

Figure S2 Operando PXRD from the LNMO cell intermittently exposed to 25 keV x-rays; a) Overview heatmap of all collected operando PXRD scans as function of time, b) operando PXRDs at selected SOC, and c) PXRDs at same selected SOC zoomed around the (511), (440) and (531) reflections of LNMO phase 1 and phase 2.

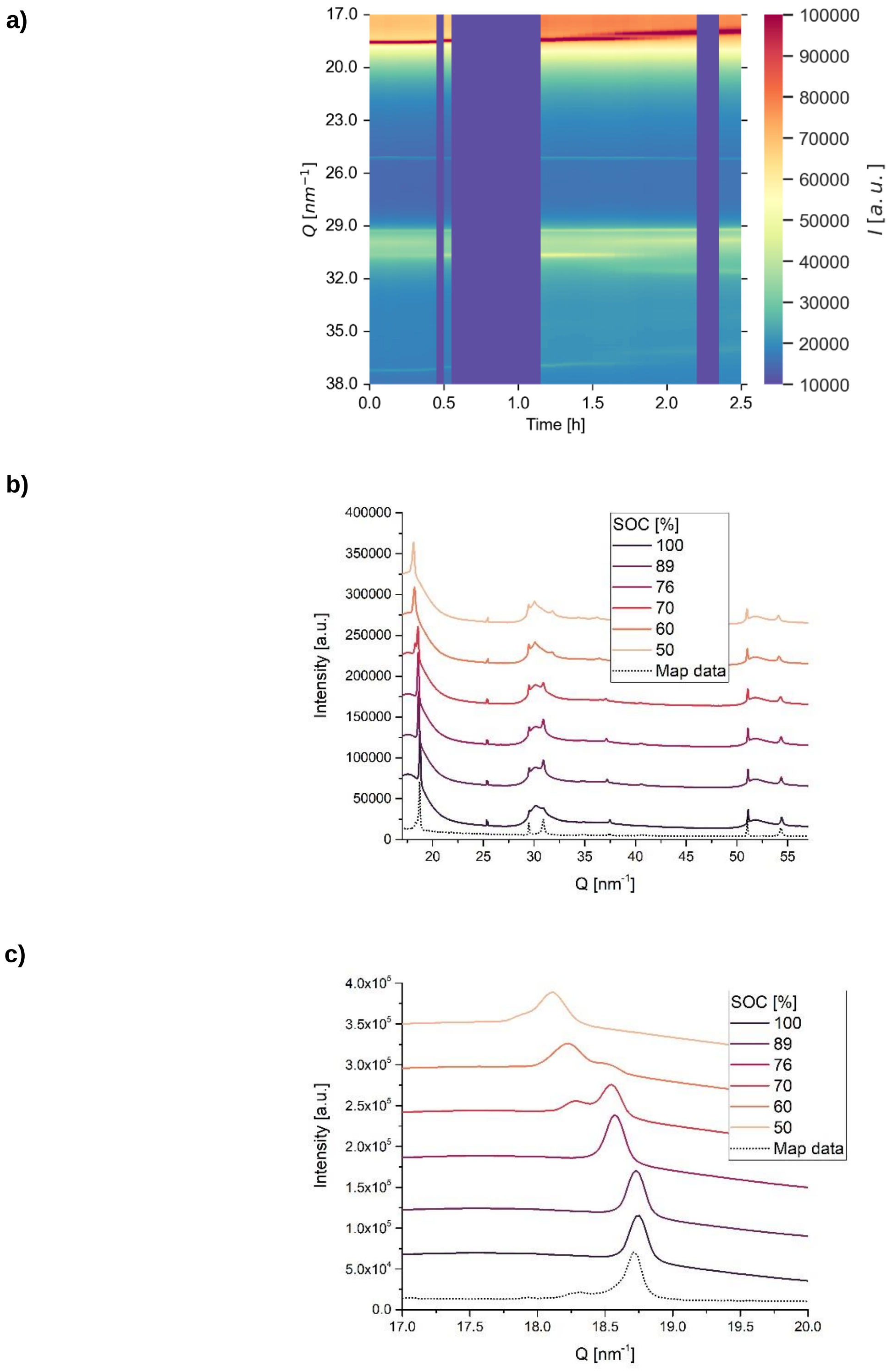

Figure S3 Operando PXRD from the Graphite half-cell intermittently exposed to 25 keV x-rays; a) Overview heatmap of all collected operando PXRD scans as function of time, b) operando PXRDs at selected SOC, and c) PXRDs at same selected SOC zoomed around the (00*l*) reflections of the graphite and Li intercalated graphite phases.

Examples of Rietveld refinements of the μPXRD mapping

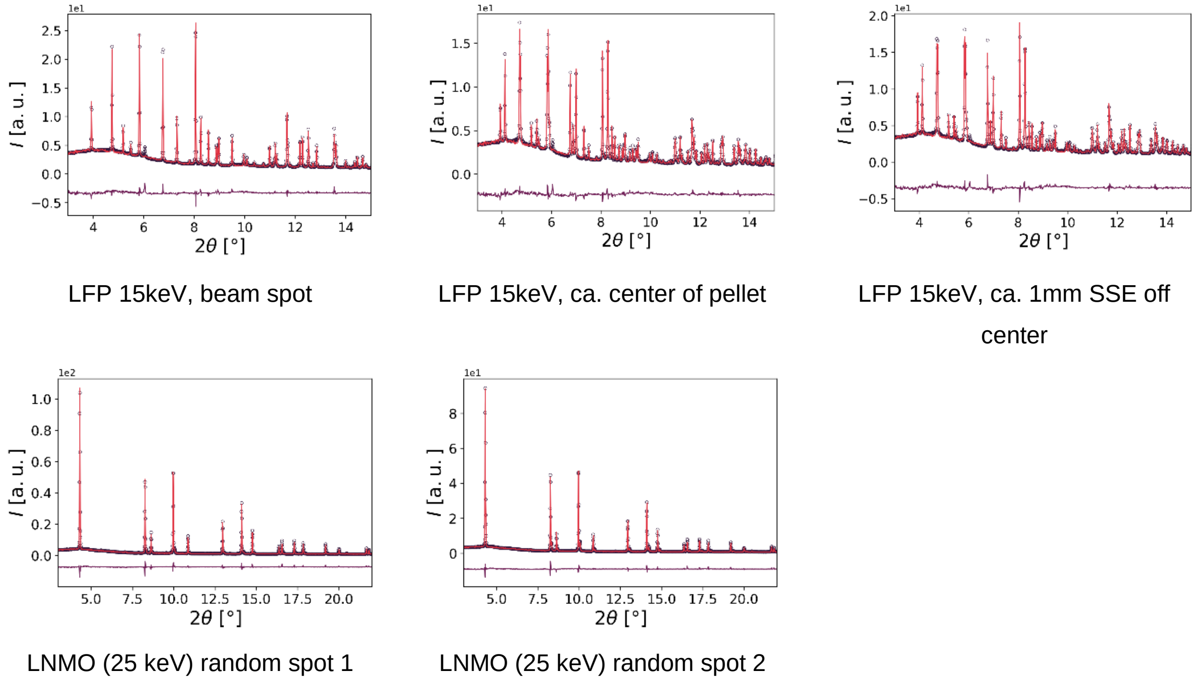

Figure S4 Examples of Rietveld refinements from selected positions.

Refined parameters for LFP electrodes

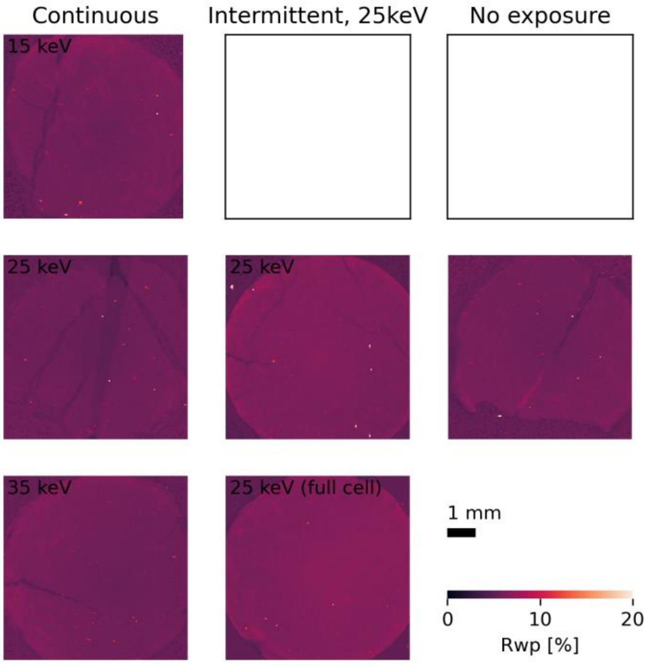

Figure S5 R_{wp} obtained from Rietveld refinements of PXRD mapping of LFP electrode pellets.

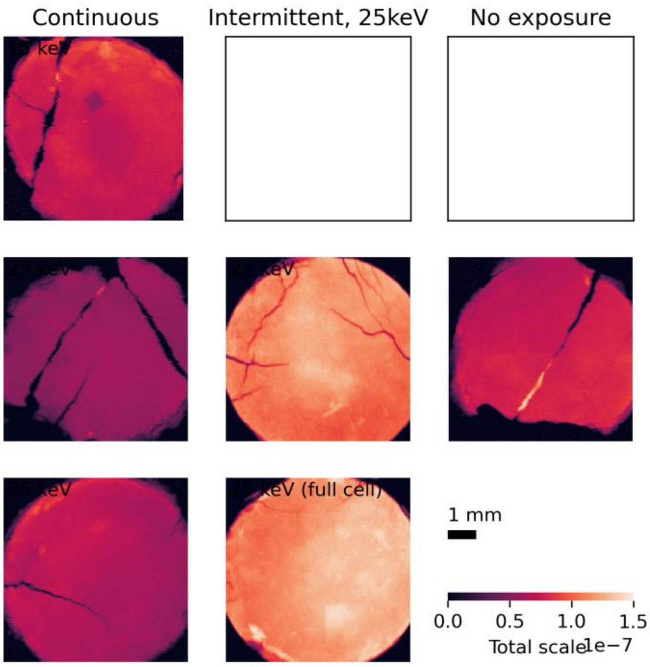

Figure S6 Total scale factors obtained from Rietveld refinements of PXRD mapping of LFP electrode pellets.

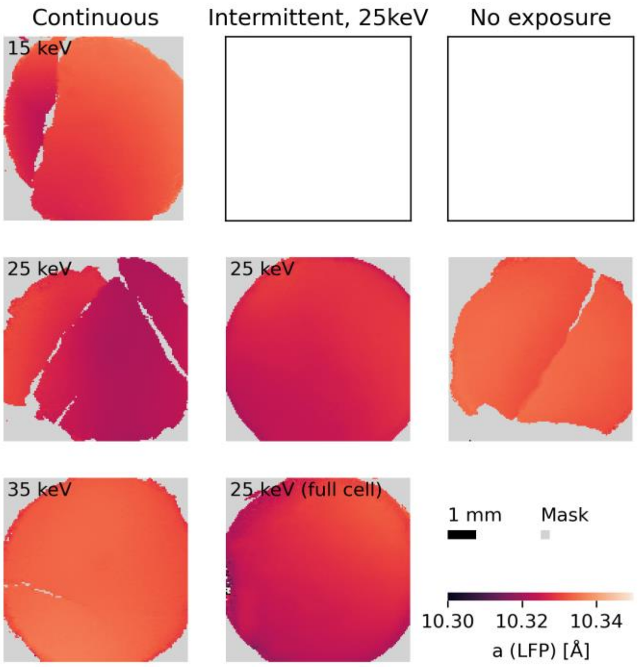

Figure S7 Lattice parameter *a* (LFP) obtained from Rietveld refinements of PXRD mapping of LFP electrode pellets.

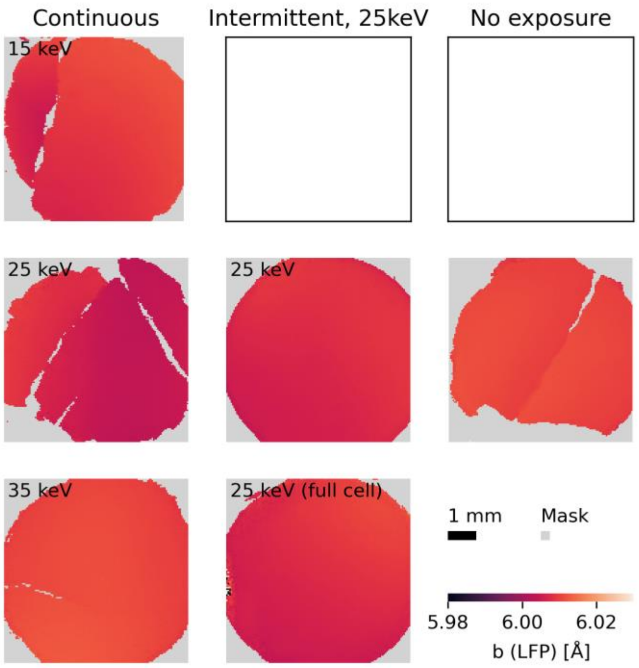

Figure S8 Lattice parameter *b* (LFP) obtained from Rietveld refinements of PXRD mapping of LFP electrode pellets.

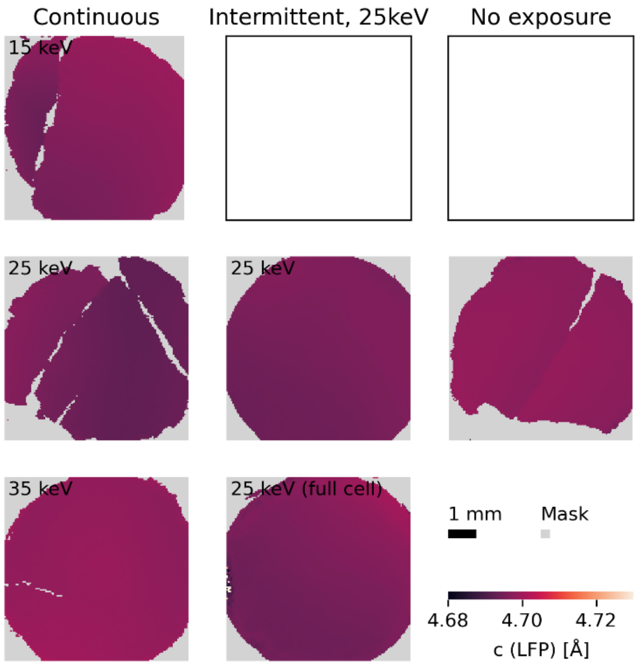

Figure S9 Lattice parameter *c* (LFP) obtained from Rietveld refinements of PXRD mapping of LFP electrode pellets.

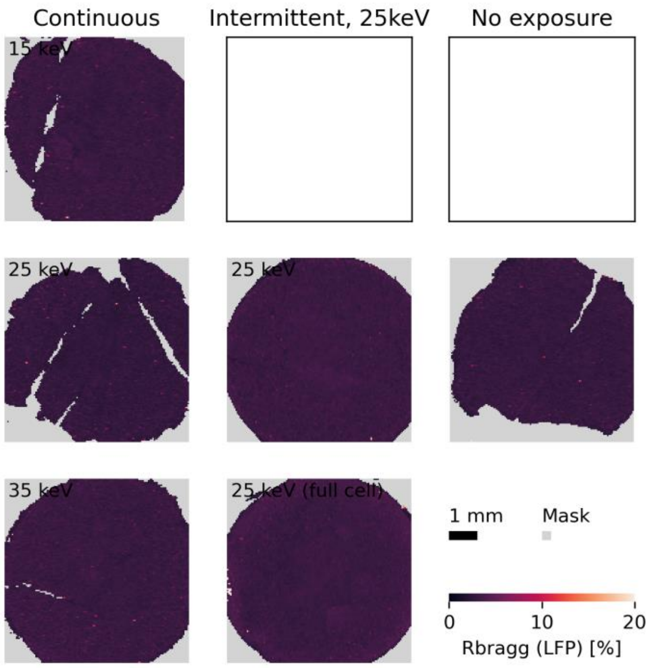

Figure S10 R_{bragg} (LFP-phase) obtained from Rietveld refinements of PXRD mapping of LFP electrode pellets.

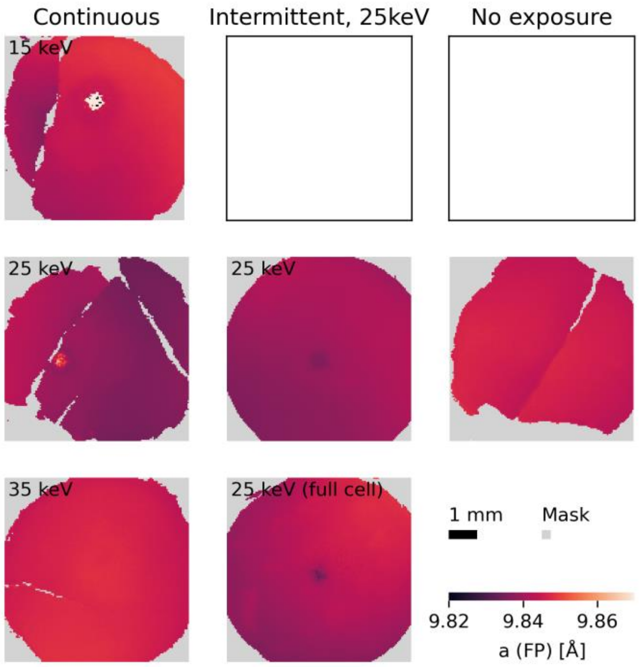

Figure S11 Lattice parameter *a* (FP-phase) obtained from Rietveld refinements of PXRD mapping of LFP electrode pellets.

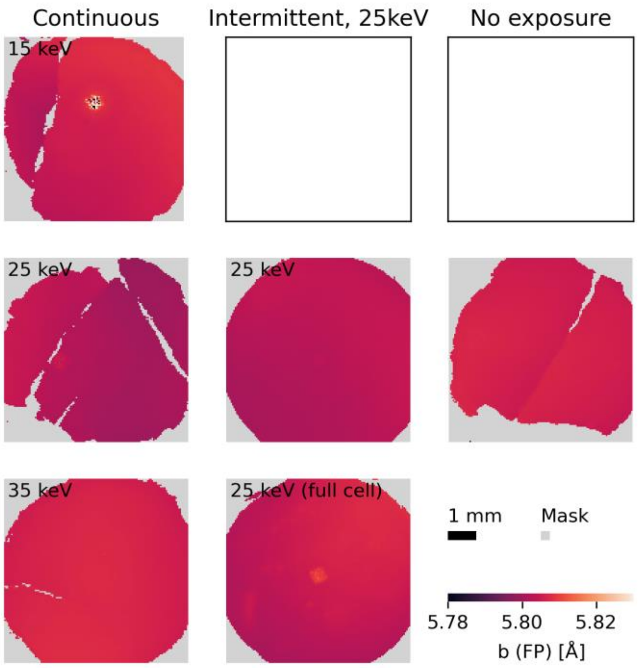

Figure S12 Lattice parameter *b* (FP) obtained from Rietveld refinements of PXRD mapping of LFP electrode pellets.

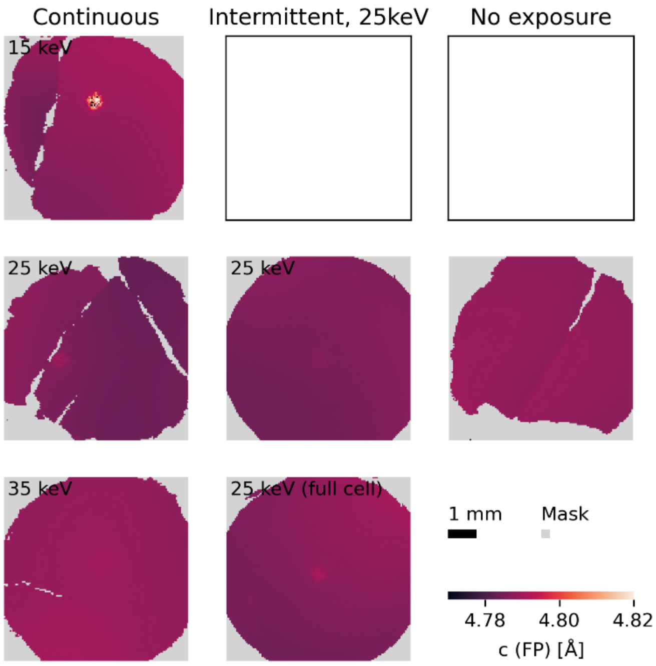

Figure S13 Lattice parameter *c* (FP) obtained from Rietveld refinements of PXRD mapping of LFP electrode pellets.

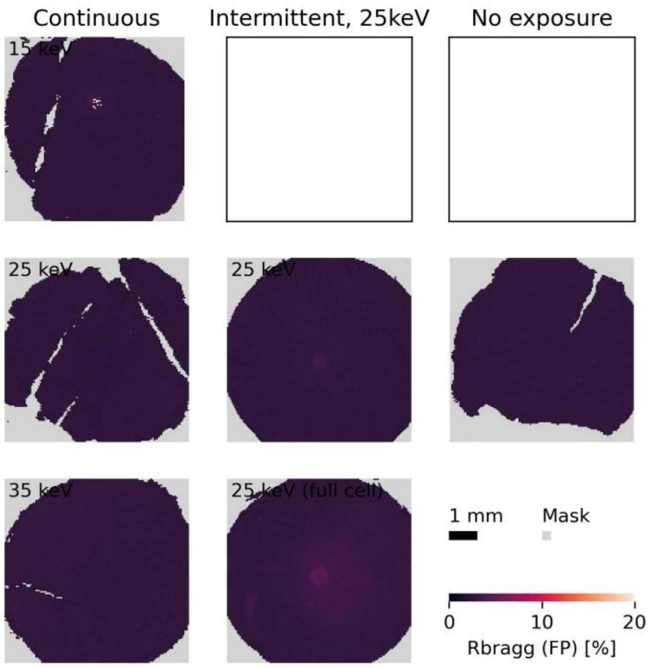

Figure S14 R_{bragg} (FP) obtained from Rietveld refinements of PXRD mapping of LFP electrode pellets.

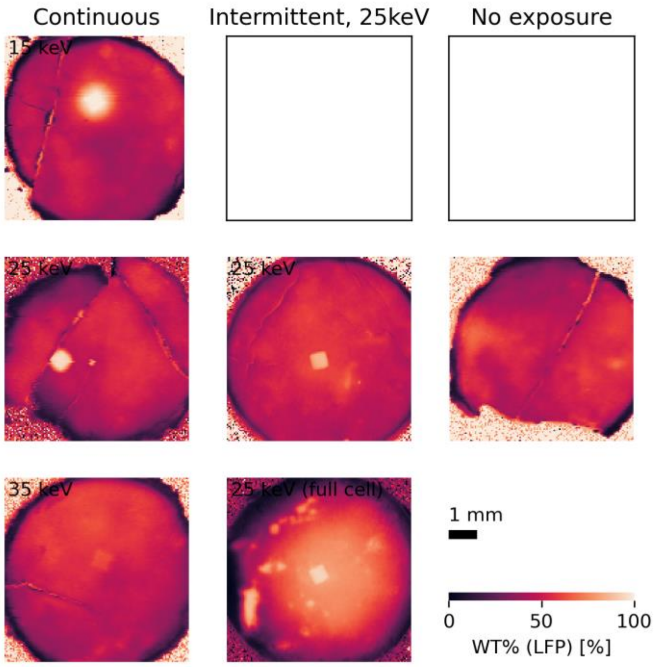

Figure S15 Weight percent of the LFP-phase obtained from Rietveld refinements of PXRD mapping of LFP electrode pellets.

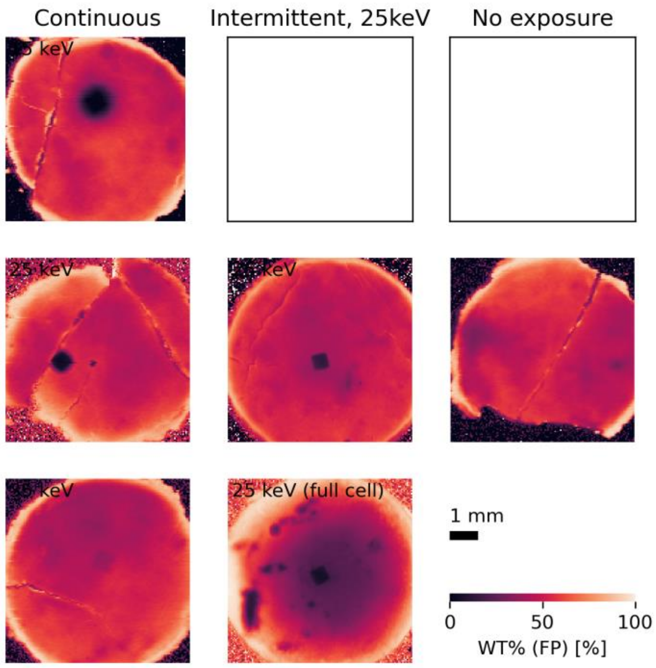

Figure S16 Weight percent of the FP-phase obtained from Rietveld refinements of PXRD mapping of LFP electrode pellets.

Refined parameters for LNMO electrodes

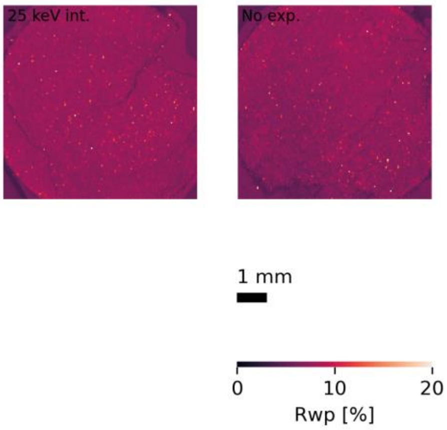

Figure S17 R_{wp} obtained from Rietveld refinements of PXRD mapping of LNMO electrode pellets.

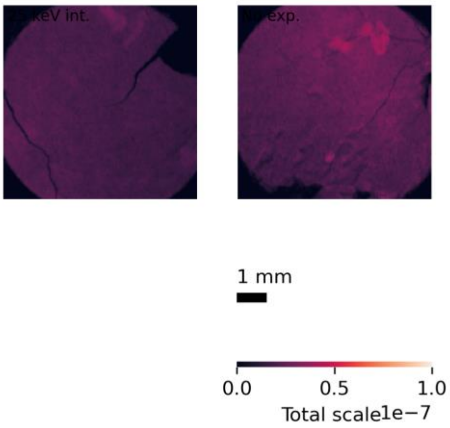

Figure S18 Total scale factors obtained from Rietveld refinements of PXRD mapping of LNMO electrode pellets.

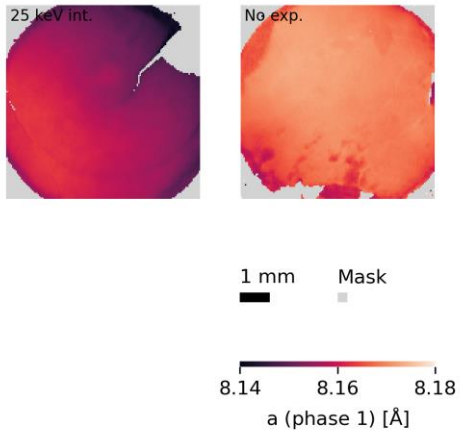

Figure S19 Lattice parameter *a* (phase 1) obtained from Rietveld refinements of PXRD mapping of LNMO electrode pellets.

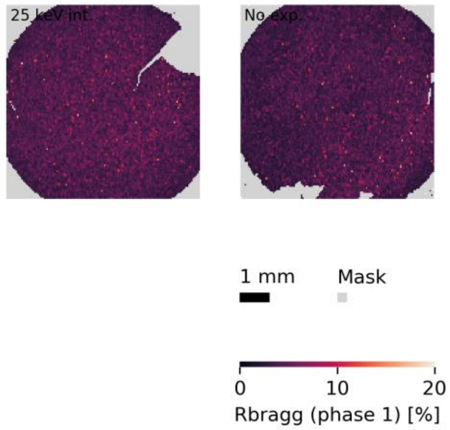

Figure S20 R_{bragg} (phase 1) obtained from Rietveld refinements of PXRD mapping of LNMO electrode pellets.

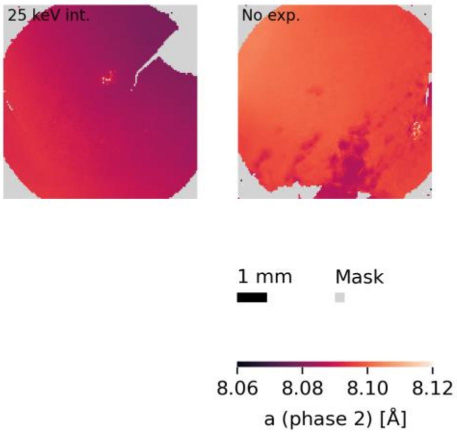

Figure S21 Lattice parameter *a* (phase 2) obtained from Rietveld refinements of PXRD mapping of LNMO electrode pellets.

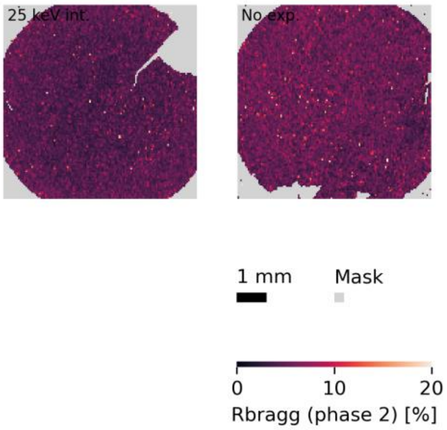

Figure S22 R_{bragg} (phase 2) obtained from Rietveld refinements of PXRD mapping of LNMO electrode pellets.

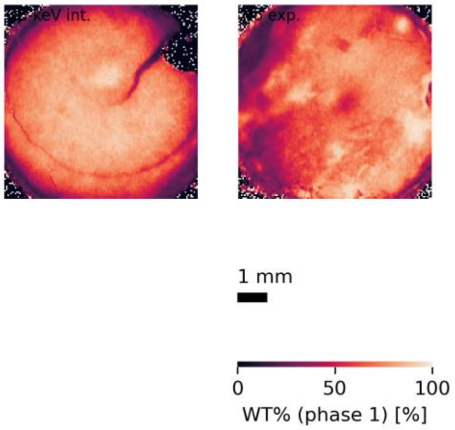

Figure S23 Weight percent of phase 1 obtained from Rietveld refinements of PXRD mapping of LNMO electrode pellets.

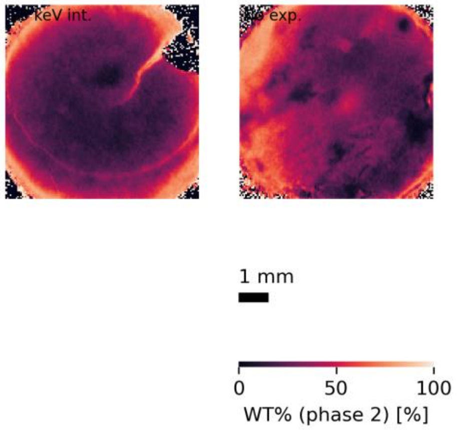

Figure S24 Weight percent of phase 2 obtained from Rietveld refinements of PXRD mapping of LNMO electrode pellets.

Zoom of SOC maps for LFP electrodes

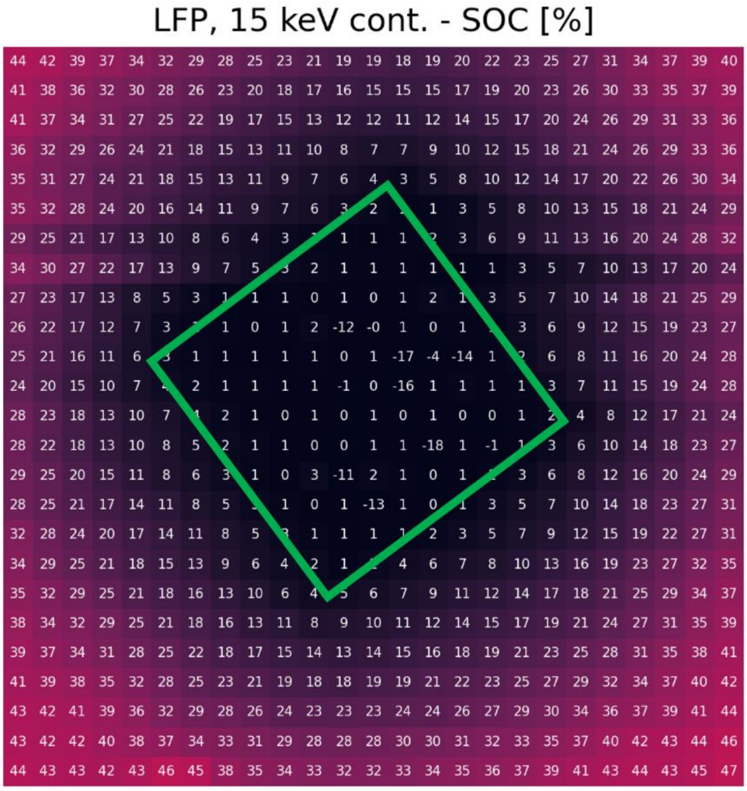

Figure S25 SOC map zoomed around beam spot with the computed SOC in % annotated in each pixel for the LFP electrode exposed continuously with 15 keV X-rays. The green box represents a 0.5 mm × 0.5 mm square illustrating the size of the beam profile.

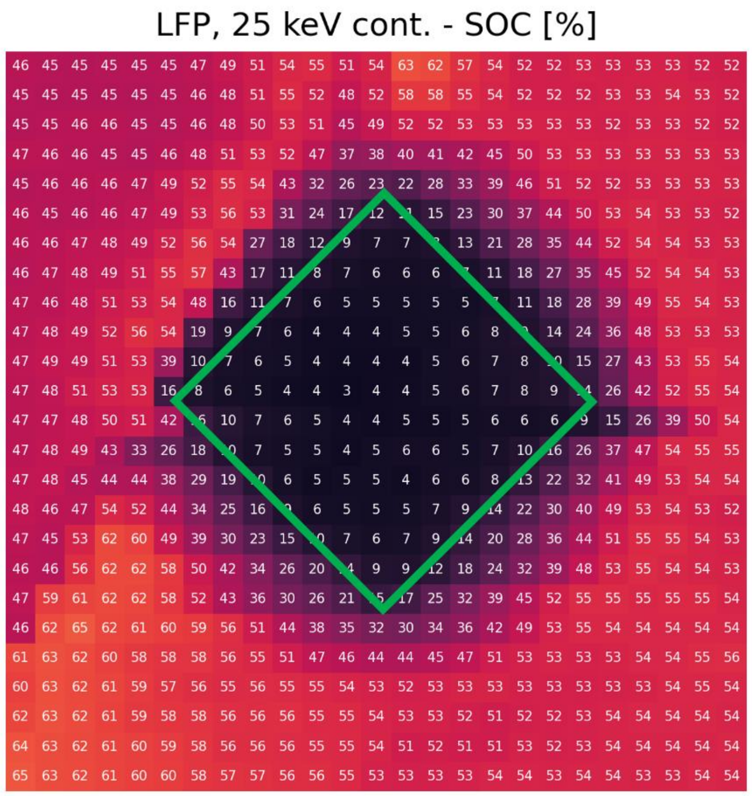

Figure S26 SOC map zoomed around beam spot with the computed SOC in % annotated in each pixel for the LFP electrode exposed continuously with 25 keV X-rays. The green box represents a 0.5 mm × 0.5 mm square illustrating the size of the beam profile.

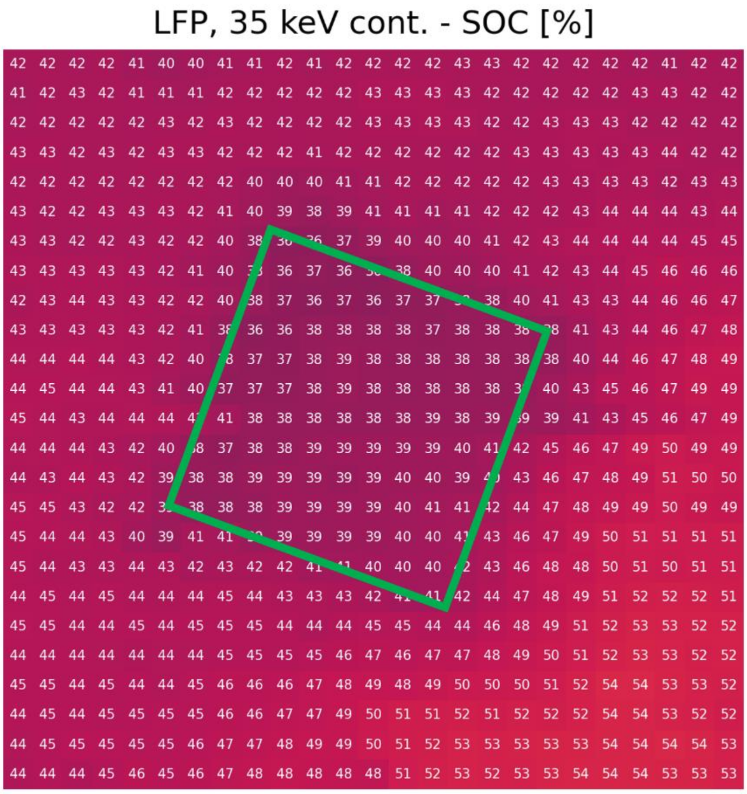

Figure S27 SOC map zoomed around beam spot with the computed SOC in % annotated in each pixel for the LFP electrode exposed continuously with 35 keV X-rays. The green box represents a 0.5 mm × 0.5 mm square illustrating the size of the beam profile.

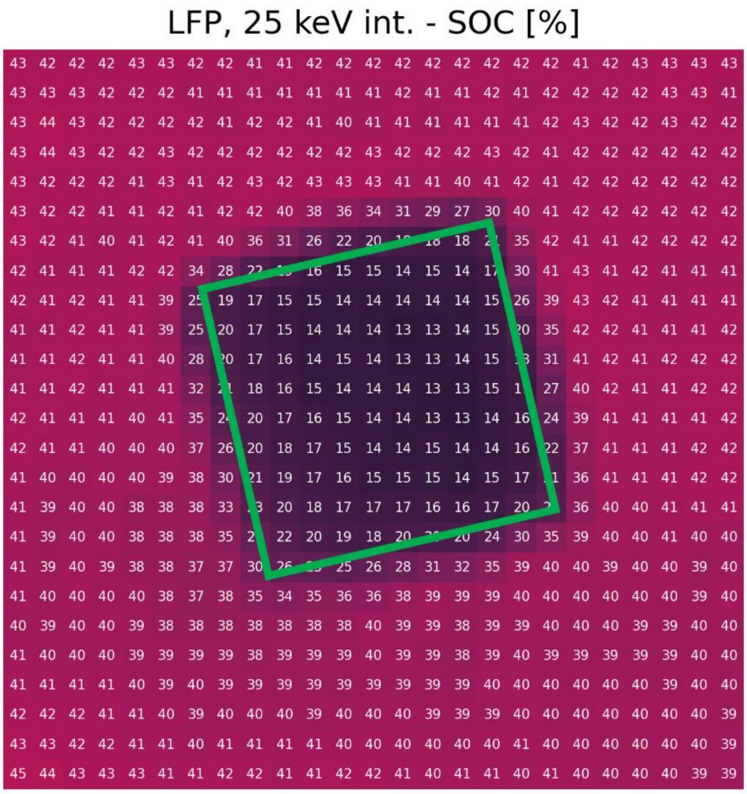

Figure S28 SOC map zoomed around beam spot with the computed SOC in % annotated in each pixel for the LFP electrode exposed intermittently with 25 keV X-rays. The green box represents a 0.5 mm × 0.5 mm square illustrating the size of the beam profile.

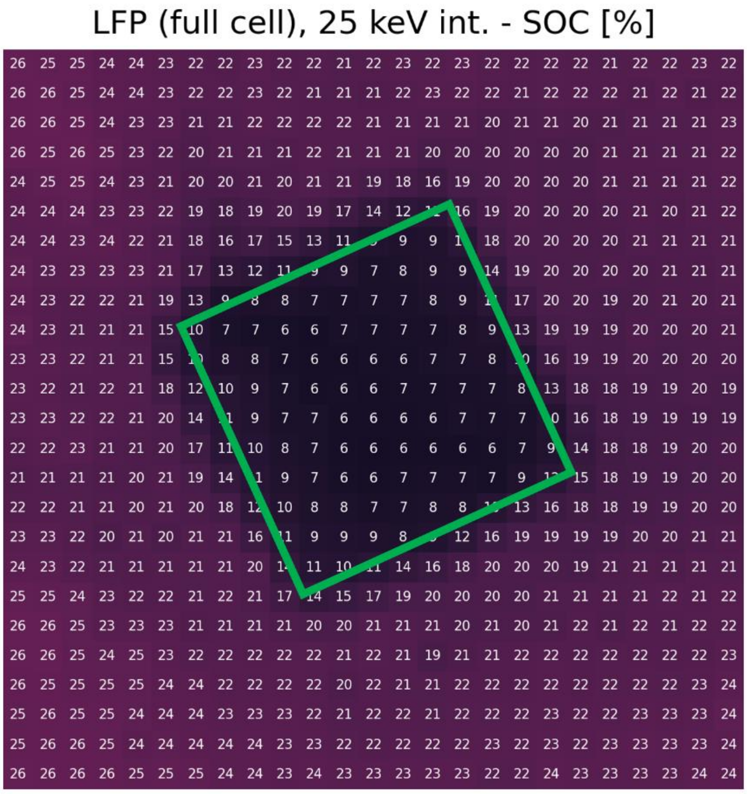

Figure S29 SOC map zoomed around beam spot with the computed SOC in % annotated in each pixel for the LFP full-cell electrode exposed intermittently with 25 keV X-rays. The green box represents a 0.5 mm × 0.5 mm square illustrating the size of the beam profile.

LNMO phase fraction and SOC relation

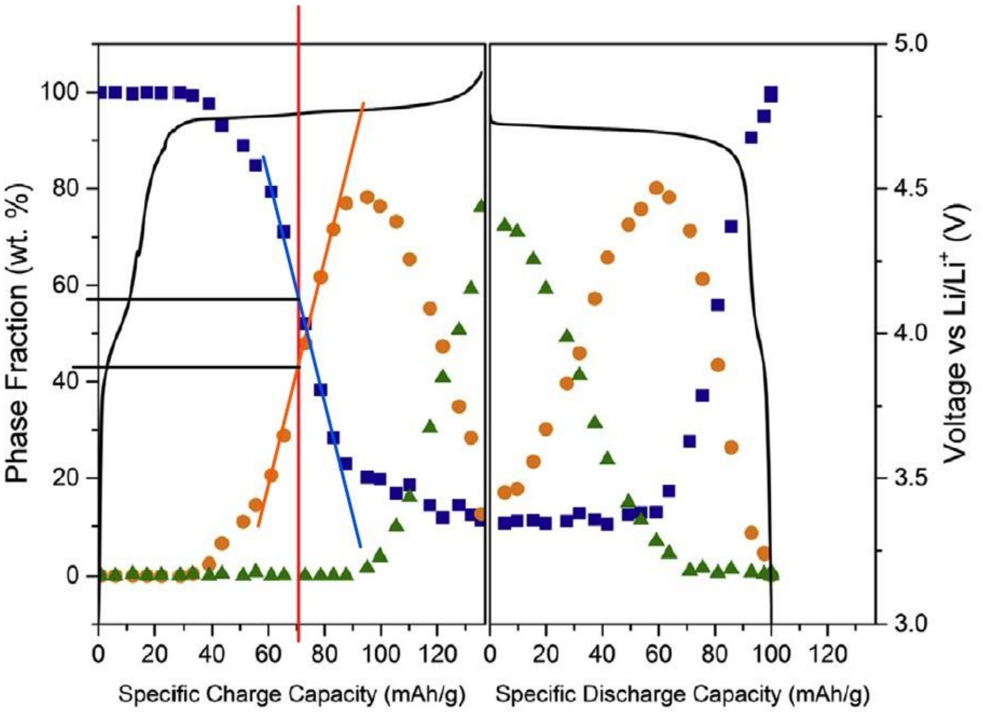

Fig. 5. Phase fraction during the first charge-discharge cycle; phase I (■), phase II (●), phase III (▲).

Figure S30 Linear regression (orange line) used for conversion of weight percent of phase 2 (named "phase II" in the reference) to SOC for LNMO in the region 35% - 70% SOC based on the paper by Samarasingha et al. (Acta Materialia, 2016)

Mass energy absorption coefficients

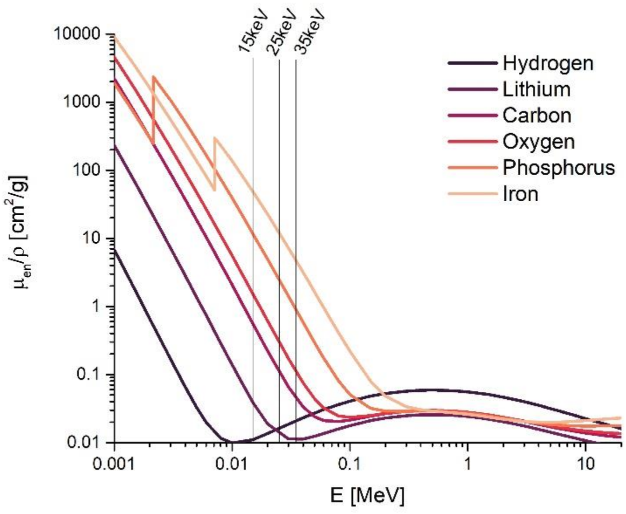

Figure S31 Mass energy absorption coefficients of the elements in an LFP electrode.

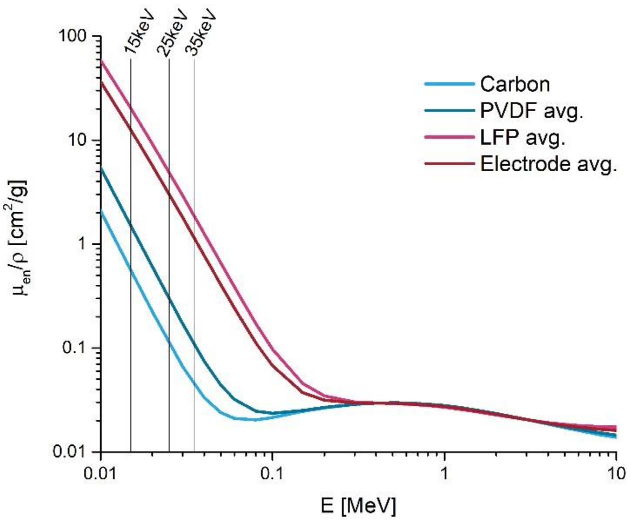

Figure S32 Calculated mass energy absorption coefficient averages of the components of an LFP electrode.

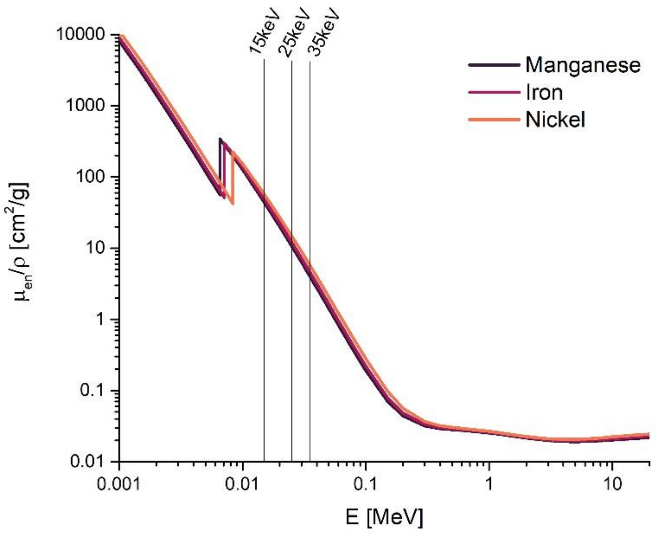

Figure S33 Mass energy absorption coefficients of selected transition metals.
